# Attentional bias modification training for insomnia: A double-blind placebo controlled randomized trial

**DOI:** 10.1371/journal.pone.0174531

**Published:** 2017-04-19

**Authors:** Jaap Lancee, Samya L. Yasiney, Ruben S. Brendel, Marilisa Boffo, Patrick J. F. Clarke, Elske Salemink

**Affiliations:** 1 Department of Clinical Psychology, University of Amsterdam, Amsterdam, the Netherlands; 2 Department of Developmental Psychology, University of Amsterdam, Amsterdam, the Netherlands; 3 School of Psychology, University of Western Australia, Perth, Australia; 4 School of Psychology and Speech Pathology, Curtin University, Perth, Australia; Charité—Universitätsmedizin Berlin, GERMANY

## Abstract

**Background:**

Attentional bias toward sleep-related information is believed to play a key role in insomnia. If attentional bias is indeed of importance, changing this bias should then in turn have effects on insomnia complaints. In this double-blind placebo controlled randomized trial we investigated the efficacy of attentional bias modification training in the treatment of insomnia.

**Method:**

We administered baseline, post-test, and one-week follow-up measurements of insomnia severity, sleep-related worry, depression, and anxiety. Participants meeting DSM-5 criteria for insomnia were randomized into an attentional bias training group (*n* = 67) or a placebo training group (*n* = 70). Both groups received eight training sessions over the course of two weeks. All participants kept a sleep diary for four consecutive weeks (one week before until one week after the training sessions).

**Results:**

There was no additional benefit for the attentional bias training over the placebo training on sleep-related indices/outcome measures.

**Conclusions:**

The absence of the effect may be explained by the fact that there was neither attentional bias at baseline nor any reduction in the bias after the training. Either way, this study gives no support for attentional bias modification training as a stand-alone intervention for ameliorating insomnia complaints.

## Introduction

Insomnia is a prevalent disorder that affects about 10% of the general population [1]. In the DSM-5, chronic insomnia is described as problems with initiating and/or staying asleep for at least three days a week for three or more months. Furthermore, these sleep problems need to have a negative effect on daytime functioning [2]. Indeed, people with insomnia report lower concentration, cognitive problems and emotional instability [3, 4]. Furthermore, insomnia is associated with psychopathology such as anxiety and depression [5]. For example, insomnia disorder is associated with a twofold chance of developing a major depression disorder in later life [6]. Apart from these psychological correlates, insomnia is also associated with somatic problems such as cardiovascular diseases [7].

Such a prevalent and impairing disorder calls for effective treatments. In general, insomnia is most effectively treated with Cognitive Behavioral Treatment for Insomnia (CBT-I). People with insomnia benefit from CBT-I, with meta-analyses reporting large treatment effects on insomnia severity [8–10]. However, not all people suffering from insomnia benefit from CBT-I. For instance, Morin and Benca [11] estimate that up to 70–80% of insomniac patients show a clinically meaningful response to treatment and only about 40% achieve clinical remission. This shows that even though the treatment is highly effective, further improving its efficacy should be a high priority.

An additional issue with CBT-I interventions is that they tend to be time and resource intensive for both clinicians and clients. Consequently, the availability of such treatments is recognized as being low in relation to need. Thus, restricted access to treatment, problems with patient motivation and adherence, and the real or perceived costs can limit the utility and uptake of such interventions [12]. While some very positive steps have been taken to increase the accessibility of CBT-I programs through online delivery [13], CBT-I nevertheless requires a sustained high level of motivation and engagement on the part of the help-seeking individual, across an extended period of time, to actively challenge the dysfunctional cognitive distortions that maintain sleep difficulties, and alter problematic patterns of behaviour.

One possible approach that may hold potential benefits to the treatment of insomnia is focusing (also) on maladaptive automatic cognitive information processes, such as selectively attending to sleep-related cues in the environment (i.e., attentional bias). Theoretically, it has been argued that attentional bias toward sleep-related information is supposed to play a key role in insomnia. For instance, Harvey’s [14] cognitive model of insomnia proposes that excessive negative cognitive activity leads to cognitive arousal and stress. This leads to selective attention (and monitoring) for negative threatening sleep-related cues. In her model, the selective attention results in a distorted perception of the sleep impairment and daytime consequences and fuels the excessive negative cognitive activity. All these factors together may cumulate into real impairment in sleep and daytime functioning and can turn into insomnia.

In another influential model, Espie and colleagues [15] further elaborate on selective attention in the context of insomnia in the attention-intention-effort pathway (A-I-E model). In this model, it is stated that sleep is an automatic process. However, this process is fragile and can be inhibited by the attention to focus on the sleep and/or directly suppress it. Espie and colleagues suggest that the sleeping system at first is threatened by selective attention toward sleep (attention), then it can be compromised by the explicit intention to sleep (intention) and eventually disrupted by a destructive combination of direct and indirect effort to sleep (effort). That is why insomnia, in this model, is seen as a ‘sleep-effort syndrome’, which is characterized by attentional bias, sleep preoccupation and mental and behavioral strategies to sleep and to avoid insomnia.

In line with these two models, it has been shown that people with insomnia show an attentional bias towards sleep-related stimuli [16–19]. However, a number of studies did not observe a sleep-related attentional bias [20–22]. In a meta-analysis, Harris and colleagues [23] recently concluded that poor sleepers have a medium to large sleep-related attentional bias compared to controls. Attentional bias thus appears to be associated with insomnia.

A viable next step seems to be testing whether the maladaptive, sleep-related attentional bias can also be modified with an attentional bias modification training (ABM) and whether this affects insomnia severity and sleep indices. Over the last years, different ABM training paradigms have been developed in the field of anxiety, whereby subjects are trained to shift their attention from threatening information (usually operationalized with pictures or words) toward neutral or positive information [24]. At first, very promising results for anxiety were observed with ABM procedures using the dot-probe task [25]. However, currently, ABM trainings for anxiety and depression are subject to more controversy with recent meta-analyses finding moderate [26] or even clinically not relevant effects [27]. Crucially, while the clinical effects of ABM have been somewhat inconsistent, the mechanistic link between changes in attentional bias and consequent changes in emotional vulnerability remains sound, with those studies failing to achieve changes in attentional bias typically also failing to observe clinical benefits [24]. Thus, it should be noted that the critical ingredient of an effective ABM training is modifying the attentional bias. A failure to manipulate the attentional bias should be interpreted accordingly and does not in itself provide evidence against the possible therapeutic value of ABM [28].

To our knowledge, there have been two studies that have sought to examine ABM training in insomnia to date, both of which have employed experimental designs with sub-clinical samples [29, 30]. Milkins et al. [30] employed a within-subjects design where 18 individuals reporting high levels of insomnia symptoms alternated completing an ABM task and a non-ABM control task before bed across six consecutive nights. While participants had no awareness of the change in tasks, they consistently reported shorter sleep onset latencies and lower pre-sleep worry on nights they had completed the ABM task compared to nights they completed the control task. In a follow-up between-subjects design, 41 students with sleep problems received five sessions of ABM or control training across as many nights. The authors showed that participants who performed the ABM training reported fewer sleep-related worries at post-test, compared to the placebo condition. However, no effects were observed for insomnia severity or sleep-related attentional bias. In addition, ABM training effects were observed on objective measures of sleep onset latency, but not on onset latency as measured with a subjective diary [29].

Overall, these studies provide some encouraging initial findings suggesting possible benefits of ABM for insomnia. As acknowledged by the authors however, a larger scale randomized controlled trial with a clinical sample is necessary in order to establish the therapeutic potential of such an intervention. Furthermore, these studies observed no changes in sleep-related attentional bias. This could have been due to a type II error (i.e., not observing an effect on attentional bias that is actually there). However, the absence of a change in attentional bias and the small sample size also raises the question whether the changes in sleep-related worry and sleep onset latency resulted from ABM or were due to other processes, including the possibility of type I errors (i.e., observing an effect that is actually not there). Thus the aim of the current study was to conceptually replicate these previous findings in a larger sample. Furthermore, we sought to determine if the effects of ABM training would be demonstrated in people meeting DSM-5 criteria for insomnia opposed to having only ‘sleep problems’ such as in Clarke et al.’s study.

Therefore, we set up a double-blind placebo controlled ABM study that differed from the Clarke study on a number of key aspects. Firstly, we included a sample of people with clinical levels of insomnia and also recruited a larger sample to ensure the study was adequately powered. Furthermore, a larger number of total training sessions were delivered over a longer period of time [31]; as such eight ABM sessions were delivered on as many training days within a two-week time frame. Lastly, we not only included a post-test but also a one-week follow-up measurement. Our hypotheses were the following: 1. Participants with insomnia will show an attentional bias towards sleep-related words at baseline. 2. Participants that receive ABM training will show a greater reduction on attentional bias compared to participants receiving the placebo training. 3. Participants that receive and ABM training will show a greater reduction on sleep complaints, sleep-related worry, and anxiety and depression measures compared to participants receiving the placebo training.

## Material and methods

### Participants

**Fig 1:**
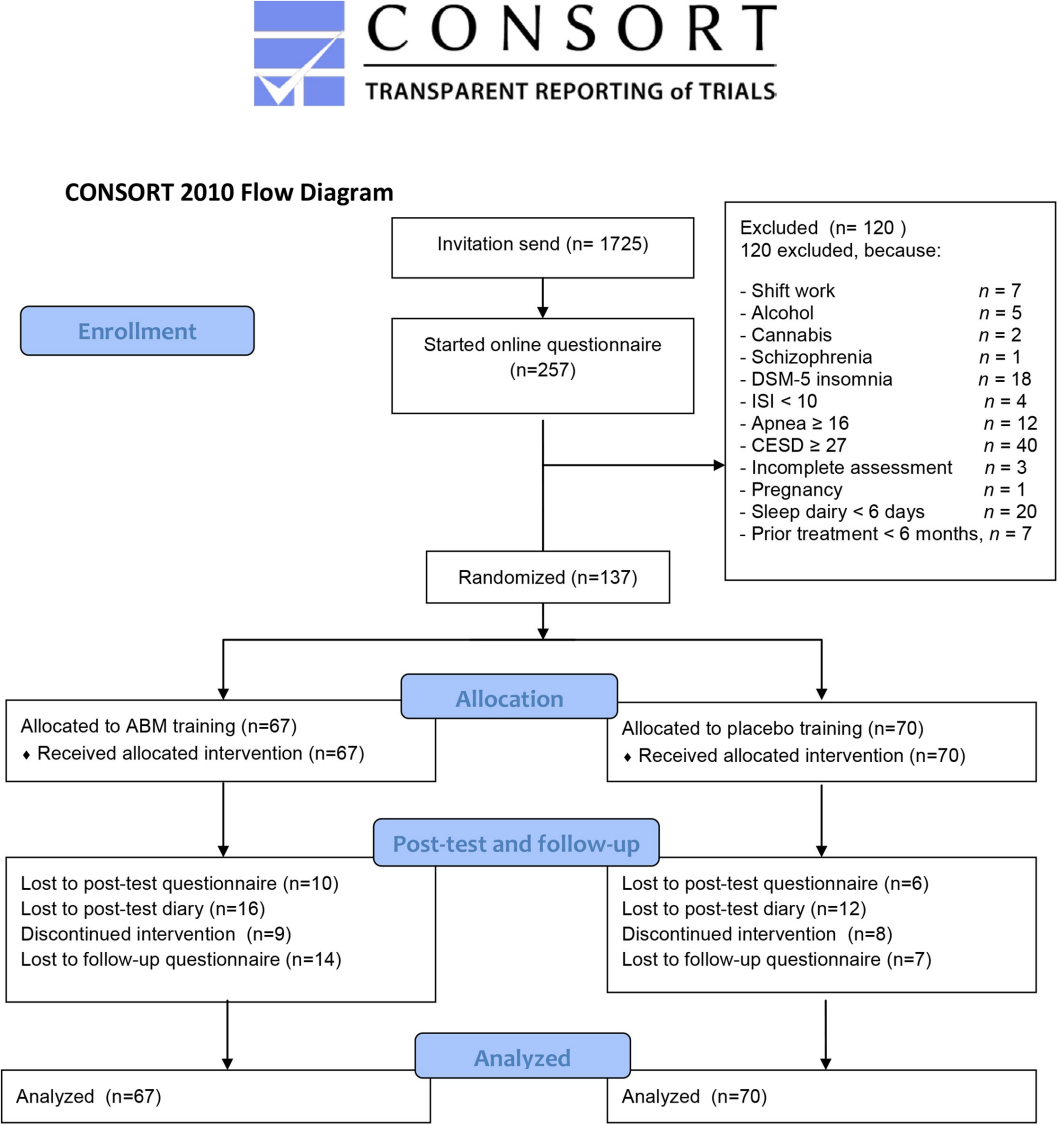
Flowchart. *Note*. For the attentional bias measurements there were 57 (active) and 64 (placebo) valid cases for the baseline. There were 39 (active) and 45 (placebo) valid cases for the post-test.

Volunteers were recruited via a popular scientific Dutch website (www.insomnie.nl). Inclusion criteria were being at least eighteen years old, meeting DSM-5 criteria for insomnia (APA, 2013) and an Insomnia Severity Index of 10 or higher (Morin, Belleville, Bélanger & Ivers, 2011). Exclusion criteria included starting a psychotherapy in the past six months, alcohol or drug abuse, high score on depressive symptoms (CES-D score of 27 or higher) [32], self-reported diagnosis of psychosis/schizophrenia, indication for sleep apnea (score of 16 or higher on the sleep apnea scale of the SLEEP-50) [33], doing shift-work, pregnancy or breastfeeding. We sent an invitation e-mail to 1725 people who earlier expressed their interest in insomnia studies. From these invitations, 257 started with the first online questionnaire. Of these, 100 were excluded from the study due to ineligibility and another 20 did not fill in sufficient days (≥6) of the sleep diary. Fig 1 provides a flowchart and numbers of participants excluded per criterion. This left us with a sample of 137 participants that were randomized into the ABM-training (*n* = 67) or ABM-placebo condition (*n* = 70). Please see Table 1 for demographics.

**Table 1:** Demographic and Clinical Characteristics at Baseline.

		Experimental		Placebo			
Age	*M* (*SD*)	48.47 (14.76)		47.72 (11.90)		*t*(135) = -0.33; *p* = .74	
		*n*	%	*n*	%		
Gender	Female	49	70.0%	51	76.1%	χ^2^ (1) = 0.65; *p* = 0.45	
Prescribed sleep medication	Yes	17	24.3%	16	23.9%	χ ^2^ (1) = 0.00; *p* = 1.00	
In psychological treatment	Yes	4	5.7%	2	3.0%	χ ^2^ (1) = 0.61; *p* = 0.68	
Living together with partner	Yes	52	74.3%	47	70.1%	χ ^2^ (1) = 0.29; *p* = 0.70	
Currently employed	Yes	56	80.0%	45	67.2%	χ ^2^ (1) = 2.91; *p* = 0.12	
Insomnia of physical origin	Yes	6	8.6%	2	3.0%	χ ^2^ (1) = 1.94; *p* = 0.28	
Years insomnia	≤1year	8	11.6%	10	14.9%	χ ^2^ (3) = 4.03; *p* = 0.26	
	1–5year	19	27.5%	25	37.3%		
	5–10year	18	26.1%	9	13.4%		
	≥10 year	24	34.8%	23	34.3%		

### Power

We based our power estimation on the meta-analysis of Hakamata and colleagues [25] on ABM training for anxiety. In this meta-analysis a mean effect size equal to Cohen’s *d* = .61 was found. Based on a t-test, a power of .80 and an alpha level of .05 (two-sided) we needed to have 44 participants per condition. However, because we were not fully confident that we could achieve a Cohen’s *d* of .61 in insomnia patients and we wanted to correct for potential drop-out, we aimed to include 25 participants extra per group.

### Measurements

#### Dot-probe task

In order to measure attentional bias towards sleep-related cues, a modified version of the dot-probe task was used and measured online [34]. The dot-probe task is a computerized speeded reaction-time task wherein two cues appear simultaneously at different locations on a computer screen. After the cues disappear, a target probe appears at the location of one of the two cues. Participants are required to respond as fast as possible to a feature of the probe. In the version of the task employed in the current study, each trial began with a fixation cross displayed on the screen for a duration randomly drawn from a uniform distribution ranging from 500ms to 1000ms. Thereafter, a negative sleep-related word and a neutral word are displayed either on the left or on the right side of the screen for 500 milliseconds (left/right side is counterbalanced), followed by an arrow (the target probe) pointing upwards or downwards displayed at the location of one of the words for 750 milliseconds. The participant is required to indicate the direction of the arrow by pressing the corresponding button on the computer keyboard (‘U’ or ‘N’; pairing of arrow direction and response button is counterbalanced across participants). The task is composed of a first practice block of four practice trials and two blocks of 96 test trials each. When participants selectively pay more attention to the sleep-related words, they will be quicker in responding to the arrow when it appears on the location of these words in comparison to the neutral words (the attentional bias). To compute the attentional bias score we subtracted the average response time when the target was presented at the location of a sleep-related word from the average response time when the target was presented at the location of a neutral-word. The higher the resulting score, the higher the attentional bias.

#### Questionnaires

At baseline, demographics such as gender and age were assessed. Furthermore, task contingency awareness in terms of the relationship between the position of the arrow probes and the words was measured at post-test.

The primary measure is the Insomnia Severity Index (ISI) [35]. The ISI contains seven items that measure the severity of insomnia complaints. Items are scored on a 0–4 Likert scale and the total ranges between 0 and 28, with higher scores indicating more severe insomnia. A score ≥ 10 is used to indicate clinical insomnia [36]. Cronbach's α is equal to .76, indicating high internal consistency. The ISI shows good construct validity since it is highly correlated with the sleep diary and polysomnography [37].

The Pittsburgh Sleep Quality Index (PSQI) measures the quality of sleep [38]. The PSQI is scored on a scale of seven components: sleep quality, sleep latency, sleep duration, sleep efficiency, sleep disturbances, use of sleeping medication and daytime functioning. Each component is rated on a 0–3 Likert scale and the total scale ranges from 0 to 21. Overall the scale has good internal consistency (α = .83) and a global PSQI score greater than 5 has high sensitivity (89.6%) and specificity (86.5%) to distinguish between good and poor sleepers [38].

Depression was measured using a Dutch translation of the 20-item Centre of Epidemiological Studies Depression scale (CES-D) [39]. The items are scored on a 0–3 Likert scale and the total ranges from 0 (no depressive symptoms) to 60 (severe depressive symptoms). A score ≥ 27 is used to indicate more severe depression symptoms [32]. This scale has good internal consistency (α = .79–.92), and the validity of the Dutch scale is comparable to that of the original version [39, 40].

Anxiety symptoms were assessed with the Dutch version of the seven anxiety items of the Hospital Anxiety and Depression Scale (HADS) [41, 42]. Items are scored on a 0–3 Likert scale and the total score ranges from 0 (no symptoms of anxiety) to 21 (severe symptoms of anxiety). The internal consistency of the HADS is good (α = .80–.84) as is the test–retest correlation (*r* = .89; *p* < .001).

Sleep-related worries were measured with the Anxiety and Preoccupation about Sleep Questionnaire (APSQ) [43]. The ASPQ consists of 10 items asking about concerns about sleep, the consequences of poor sleep and about control of sleep. Items are scored on a scale from 1 (strongly disagree) to 5 (strongly agree) and the total score ranges from 10 to 50 (Jansson-Frojmark, Harvey, Lundh, Norell-Clarke, & Linton, 2011). The internal consistency of the APSQ is excellent (α = .93) and the APSQ correlates significantly with pre-sleep cognitive arousal and sleep-related beliefs [44].

Sleep-related dysfunctional beliefs were assessed with the Dutch translation of the 16-item brief Dysfunctional Belief and Attitudes about Sleep scale (DBAS) [45]. Items are scored on a 0–10 Likert scale and the sum of the DBAS score is averaged so that the total score ranges from 0 (no dysfunctional beliefs) to 10 (severe dysfunctional beliefs). The DBAS has good internal consistency (α = 0.79) and correlates significantly with self-report measures of insomnia severity, anxiety, and depression.

#### Sleep diary

We used a Dutch translation of the consensus sleep diary [46]. This diary was kept throughout the four weeks of the study (28 days). In the diary, participants recorded time to bed, time they tried to go to sleep, time of final awakening, time out of bed, sleep onset latency (SOL), wake after sleep onset (WASO), amount of awake time between the final awakening and the time of getting out of bed/ terminal wakefulness (TWAK), number of nightly awakenings, sleep quality (1 = “very bad” to 5 = “very good”), and use of sleep medication. From these variables, the time in bed (TIB = final arising time—time to bed), total sleep time (TST = TIB–SOL–WASO–TWAK), sleep efficiency (SE = [TST/TIB] × 100), and total wake time (TWT = SOL + WASO + TWAK) were calculated.

#### Intervention

The ABM training was delivered by means of an adjusted version of the dot-probe assessment task [47]. When the dot-probe is adapted to training the stimulus-response contingency is manipulated in order to train participants to shift attention away from the negative sleep-related stimulus towards the neutral stimulus, thus reducing their attentional bias towards sleep cues [48]. Thus, in the training condition, the target arrow is always displayed in the same location previously occupied by the neutral word, thus encouraging selective attention away from the sleep-related negative word. In the placebo condition, the distribution of the placement of the arrow is 50–50, similarly to the assessment version of the task. Each training session takes approximately 15 minutes. The training task starts with a block of eight practice trials followed by two training blocks of 144 trials each, for a total of 2304 training trials across the 8 training sessions.

For the ABM training we used two word sets: set A and set B, both consisting of 24 word pairs. To assess generalization to new words, different sets were used in the pre- and post-training assessment. If attentional bias at pre-test was measured with set A, the same set was used for the ABM placebo or training and set B was used for the post-test attentional bias assessment. The order of these sets was counterbalanced across participants. Sleep words were based on the words used in Clarke and colleagues [29] and MacMahon, Broomfield and Espie [49]. Because we wanted to use 48 word-pairs we also added several worry and sleep-related words (Please see S1 Table for an English translation of the words).

The neutral words were matched on the number of times a word appears in Dutch subtitles since Dutch word frequencies in subtitles is a better predictor than frequencies based on written sources [50]. For this purpose, the search engine SUBTLEX -NL was used (http://crr.ugent.be/subtlex-nl). In addition, the words were matched on the number of syllables.

### Procedure

The procedure of this study was registered at www.trialregister.nl (NTR5020) and approved by the Ethical Review Board of the University of Amsterdam. Recruitment took place in March 2015. After people showed interest in the study they gave informed consent via an online questionnaire platform (www.qualtrics) where the consent form was stored (this consent procedure was approved by the Ethical Review Board of the University of Amsterdam). After people gave their consent they filled in an online screening questionnaire. Through this questionnaire, inclusion/exclusion criteria were checked and baseline measurements were then administrated (see Fig 1 for an overview of the participants’ flow). After the baseline assessment, people filled in a sleep diary for a week for which they received e-mails each day at 5 AM, and if applicable a reminder at 11 AM. If they completed at least six out of seven days of the diary they were included in the study and were provided with a link to the online ABM training. After people logged on to the training online platform they were automatically randomized into the active or placebo condition, stratified by gender. A computerized randomization algorithm assigned participants to the condition to which the fewest participants of their gender had been so far assigned. Both the researchers and the participants were blind to the condition. After randomization, participants completed the assessment version of the dot-probe task to measure attentional bias before training. Thereafter, the participants started with the first training session. In total there were eight sessions and each session lasted approximately 15 minutes. These sessions were conducted in the second and third week of the study, from Monday to Thursday. On these days an invitation e-mail for the training was sent at 7 PM and a reminder at 10 PM. Participants were asked to carry out the training between 7 PM and 11 PM. In case participants missed the training session, they could catch up with it during the weekend.

The post-test assessment took place the day after the two-week training was completed. Regardless of the amount of training sessions completed, the post-test questionnaires were always administered two weeks after completing the first week of the sleep diary. The follow-up questionnaires were administered a week after completing the post-test questionnaires. If people did not fill in the post-test questionnaires, a reminder was sent two days later (and as a consequence the follow-up was also two days later). The sleep diary was filled in throughout the four weeks; it started after the baseline questionnaire and stopped together with the follow-up questionnaire. After completion of the last follow-up questionnaire, participants were debriefed about the goal of the study and people in the placebo condition got the opportunity to complete the real ABM training. As a token of gratitude, all participants received an online or paper version of the self-help treatment materials described in Lancee, Spoormaker, van Straten, and van den Bout [51].

### Statistical analyses

Before running the main analyses, dot-probe task data was pre-processed to limit the influence of outlying data. Trials with incorrect responses or latencies less than 200ms or greater than 2000ms were removed before computing the attentional bias score. Task split-half reliability was computed via a bootstrap procedure, by randomly splitting the total amount of trials in two halves, such that each half had the same number of congruent (i.e., probe at the location of the sleep-related word) and incongruent trials (i.e., probe at the location of the neutral word). Next, attentional bias scores were computed for each half and the correlation between these halves was calculated across participants. The procedure was repeated 100 times and an average split-half correlation was computed. Test-retest reliability was also computed by correlating baseline and post-test attentional bias scores.

All analyses were carried out on the intention-to-treat principle with multilevel regression analyses. Multilevel regression enables the retention of all cases in the analyses (also the ones that only filled out the pre-test) [52]. In the model, we added ‘time’, ‘condition’, and ‘time x condition’ as main predictors, with the interaction effect as the main variable of interest. Before running the multilevel regression models, we checked for possible randomization differences at baseline and we found no significant differences between conditions (all *p* values > .09). Furthermore, we used *t*-tests and chi-squares to check whether there were variables associated with non-response (i.e. not responding to post-test questionnaires) in either of the condition. In the ABM condition, the following variables were negatively associated with non-response at post-test and follow-up: PSQI and amount of training sessions completed, suggesting that higher symptom levels and greater engagement in task completion were both associated with retention in the treatment program. Terminal wakefulness was positively related to non-response (i.e., higher terminal wakefulness indicated greater non-response rate at post-test). In the placebo condition, the following variables were negatively associated with non-response at post-test and follow-up: CESD, HADS-A, and amount of training sessions completed. Number of awakenings was positively related to non-response (i.e., a higher number of awakenings was associated with greater non-response rate). If any of these variables correlated with the dependent variables they were added as a covariate in the multilevel regression models.

Because we had some missing values at post-test and follow-up, we also used a predictive mean matching procedure to impute ten separate datasets [53]. The average of these datasets was used for the missing values. In the tables the means are based on these imputed values. In addition, we also report results for treatment completers who completed at least six out of the eight training sessions. For these completers we also imputed the missing values based on multiple imputation.

Within-group Cohen’s *d* effect sizes were calculated with: *d* = *M*_pre_−*M*_post_/*SD*_pooled_, in which *SD*_pooled_ = √ ([*SD*_pre_^2^ + *SD*_post_^2^] / 2). Between-group effect sizes were calculated with: *d* = ([*M*_pre1_ –*M*_post1_]–[*M*_pre2_ –*M*_post2_]) / *SD*_change_, in which *SD*_change_ is the pooled *SD* of the pre–post change score. For all the multilevel models we checked whether the residuals met the assumption of normality. Throughout the study we used an alpha level of .05 (two-sided).

Outlying scores on the main outcome variables were removed if they had a baseline z score of 3.29 or higher. Three full diaries (baseline and post-test) in the placebo condition were deleted because participants reported in their baseline diary that they did not sleep for a full week. Furthermore, we removed the following outliers from the baseline and post-test: one attentional bias score and one number of awakenings score in the placebo condition; one anxiety, one sleep onset latency, one terminal wakefulness score, and one number of awakenings score in the experimental condition.

## Results

### Number of training sessions completed

Of the participants in the ABM-training condition, 58 (86.6%) completed six or more training sessions and on average they completed 7.10 (*SD* = 2.05) sessions. Of the participants in the ABM-placebo condition, 62 (88.6%) completed six or more training sessions and on average they completed 7.29 session (*SD* = 1.76), *t*(135) = .56, *p* = .58.

### Hypothesis 1: Attentional bias prior to training

A paired-sample *t*-test showed that the average response time on probe on the sleep-word (*M* = 720.17, *SD* = 131.70) was not significantly lower than probe on the neutral word (*M* = 720.90, *SD* = 131.78), *t*(1, 123) = 0.30, *p* = .77. This means that, contrary to our first hypothesis, there was no indication of an attentional bias towards sleep-related word cues in people with insomnia. Split half reliability of the dot-probe task at baseline was *r* = -.06, *p* = .54 and test retest reliability was *r* = -.09, *p* = .43.

### Hypothesis 2: Efficacy of the training on attentional bias

The multilevel regression analysis showed that there was neither significant time effect nor an interaction effect (time × condition) on attentional bias scores at post-test. This means that, contrary to our second hypothesis, the training was not more (nor less) effective in ameliorating attentional bias than the placebo training. Cohen’s *d* effect size was equal to .01. In Table 2 means, standard deviations and Cohen’s *d* of attentional bias scores can be found. See S4 Table for the multilevel regression analysis. The completers’ sample showed the same pattern as the imputed sample (S3 Table).

**Table 2:** Baseline, Post-test, and Follow-up Scores and Cohen’s *d* Effect Sizes for the Experimental and Placebo Conditions.

Study variable	Condition	Baseline	Post-test	Follow-up	Cohen’s *d*		
		Mean (SD)	Mean (SD)	Mean (SD)	Within-group baseline-post-test	Within-group baseline-follow-up	Between-group post-test	Between-group follow-up
Ins0.00omnia Severity (ISI)	Experimental	14.39 (3.00)	12.96 (3.88)	12.80 (5.21)	-0.41***	-0.37***	0.00^ns^	0.10^ns^
	Placebo	14.73 (3.02)	13.29 (3.48)	13.58 (3.98)	-0.44***	-0.32***		
Depressive symptoms	Experimental	14.63 (6.68)	16.11 (7.93)	14.48 (8.60)	-0.22^ns^	-0.02^ns^	0.07^ns^	0.22^ns^
(CESD)	Placebo	15.44 (5.45)	17.42 (7.85)	17.15 (8.81)	-0.29*	0.23^ns^		
Anxiety (HADS)	Experimental	6.11 (3.09)	5.55 (3.77)	5.69 (3.94)	-0.16*	-0.12**	0.21^ns^	0.13^ns^
	Placebo	5.73 (3.15)	5.83 (3.15)	5.81 (3.68)	0.02^ns^	0.01^ns^		
Sleep worry (APSQ)	Experimental	35.52 (7.40)	31.04 (9.38)	29.89 (10.45)	-0.53***	-0.62***	0.05^ns^	0.17^ns^
	Placebo	36.03 (7.15)	32.07 (9.50)	32.03 (10.00)	-0.47***	-0.46***		
Sleep onset latency	Experimental	41.78 (36.76)	41.38 (32.78)	-	-0.01^ns^	-	0.10^ns^	-
(SOL)	Placebo	36.98 (28.04)	39.21 (30.42)		-0.10^ns^			
Sleep efficiency (SE)	Experimental	68.96 (15.66)	70.88 (13.26)	-	0.14^ns^	-	0.09^ns^	-
	Placebo	69.10 (14.75)	69.90 (14.41)		0.06^ns^			-
Attentional bias	Experimental	3.97 (31.64)	4.77 (21.16)	-	0.03^ns^	-	0.01^ns^	
	Placebo	-0.99 (25.16)	0.12 (21.28)		-0.05^ns^			

*Note*. Means, *SD*’s and *d*’s are based on the imputed sample. Please see S2–S4 Tables for the remaining variables, the completers’ sample and the multilevel regression coefficients.

* = *p* < .05

** = *p* < .01

*** = *p* < .001

ns = not significant.

### Hypothesis 3: Efficacy of the training on the outcome measures

The multilevel regression analyses showed that there were significant time effects at post-test for both conditions on the ISI (primary measure), the PSQI, and total sleep time. Significant time effects were found for the CESD only on the ABM condition and for the HADS-A and TWAK only for the placebo condition. For the remaining variables no time effects were observed. Furthermore, no significant interaction effects (time × group) were found on any of the variables at either the post-test or follow-up. This means that, contrary to our third hypothesis, the training was not more (nor less) effective than the placebo training. Cohen’s *d* effect sizes ranged between .00 to .40. In Table 2 means, standard deviations and Cohen’s *d* for attentional bias, Insomnia Severity Index (ISI), Sleep efficiency, Sleep onset latency, depressive symptoms (CESD), anxiety symptoms (HADS-A), and worry about sleep (APSQ) can be found. For the remaining variables, the completers’ sample and the multilevel regression coefficients please see S2–S4 Tables. In the completers’ sample the same pattern of results was observed as in the imputed sample.

### Treatment awareness

Of the 60 participants that filled out the post-test in the placebo condition, seven thought there was a relationship between the arrow and the words; four of these recognized the correct relationship. Of the 54 participants in the experimental condition, 12 thought there was a relationship between the words and the arrow; however, none recognized the correct relationship.

### Adverse events

There were no adverse events reported in this trial.

## Discussion

We investigated whether a multiple-session ABM training could reduce sleep-related attentional bias and sleep complaints in a sample of 137 people with insomnia. In this placebo controlled randomized study we found no indication for the presence of a sleep-related attentional bias and no evidence that we could change the bias or the sleep complaints. We did observe a general reduction in both groups on insomnia symptoms, which is most likely due to non-specific factors such as keeping a diary and taking part in a research trial.

The absence of a sleep-related attentional bias at baseline is not in line with earlier research that did evidence an attentional bias toward sleep in individuals with insomnia [16–19], nor is in line with the models of Harvey [14] and Espie et al. [15] where it is assumed that attentional bias plays a role in insomnia. However, the findings are in agreement with three other studies that could not demonstrate this bias using the Stroop task [20–22] and with the first ABM study that also could not detect an attentional bias using the dot-probe [29]. Our failure to change attentional bias and sleep problems using ABM could be related to the absence of an attentional bias at baseline as studies have shown larger ABM effects in individuals with higher attentional bias at baseline [54, 55]. On the other hand, there have been reports of samples without an attentional bias at baseline where ABM still reduced attentional bias as well as anxiety symptoms (e.g. [56]).

At any rate, changing attentional bias is a critical issue since it is the alleged working mechanism of ABM interventions. Furthermore, only when attentional bias is changed, effects on outcome measures are observed in the field of anxiety [28]. In the current study, we could not observe any change in attentional bias, which is in line with other research on sleep-related ABM [29, 30]. The poor psychometric properties of the dot-probe task may provide a possible valid reason for not observing these changes in bias (which we discuss below). Here, we want to stress that, if this bias is not changed, it is difficult to draw conclusions on the beneficial effects of changed attentional bias on outcome measures (i.e., causal relationship) and on the efficacy of ABM in general.

In contrast to the study of Clarke and colleagues [29], we did not observe significant interaction effects (time × group) on any of the outcome variables. Clarke and colleagues [29] did observe stronger reductions in sleep-related worry and objective sleep onset latency in the ABM group compared to a placebo condition, but did not find any reduction in insomnia severity and self-reported sleep onset latency. As we pointed out in the introduction, these effects could have been a result of a type-I error (i.e., detecting an effect that is not present). Since our study was more adequately powered, a type-II error is not a probable explanation for the absence of the effect in our study. Furthermore, our results seem more consistent with the working mechanism of ABM [57]: no change in attentional bias is associated with no change in symptoms, whereas observing symptom reductions after no change in attentional bias [29] is deviating from contemporary models regarding ABM’s mechanism of change.

Another explanation may be that we did not succeed in manipulating the bias, whereas the studies by Clarke and colleagues [29], and Milkins and colleagues [30] did, albeit failed to detect it (i.e., a type-II error—not detecting an effect that actually is present). A subsequent logical question is then “what could explain the absence of effect in our study and the observed effects in these prior studies?” Overall we think that the ABM procedures between the two studies were highly similar. Therefore, we will now first review the strengths and limitations of the current study in order to shed more light on the extent that our study should be seen as a manipulation failure (i.e., not being able to change the attentional bias) or as evidence against our hypotheses (i.e. ABM training does not lead to improvements in insomnia patients).

In our view, this study had several strengths. First, as we mentioned, the study was adequately powered to find an average effect size difference (*n* = 137 in our study, versus *n* = 36 in [29]); therefore, we are confident that the null findings in this study were not due to a lack of power. Second, we used a randomized placebo controlled double-blind design and we followed CONSORT guidelines [58]. Furthermore, we included people who suffered from insomnia based on DSM-5 criteria, which is more ecologically valid than testing students or good sleepers. In addition, we used a naturalistic design by deploying the ABM training online so people could do the training at home (but did not do so with a smartphone app as Clarke and colleagues [29]). However, the naturalistic design is also a limitation of the study since this meant that we had no optimal control over the training (e.g., time of administration, computer screen sizes, etc.). This may be of especial importance for the online measurement of the dot-probe. The dot-probe is a timed task that may be susceptible to variances in internet speed and/or precision of computer timer system (although several studies have reported similar effects of studies delivered at home compared to those delivered in the lab [59–61]). For instance, Carlbring and colleagues [62] found that a previously effective offline study on social anxiety was not successfully translated to an online format.

There were also some (other) limitations to this study. The most important limitation regards the psychometric properties of the attentional bias measurement task (dot-probe). As we mentioned above, we detected neither attentional bias at baseline nor any change of attentional bias. Recently it was shown that the dot-probe task lacks reliability [63, 64]. For instance, Waechter and colleagues [64] found Cronbach’s alpha’s for attentional bias scores in the range of .03–.25. The same pattern was found in our sample with a split-half reliability of *r* = -.06. This means that the (change in) attentional bias may have been present in our sample, but the dot-probe may have failed to detect it. This poses a serious problem since now we cannot be certain whether we did not succeed in changing the attentional bias or we could not detect this change. Future studies should employ a more reliable measure of attentional bias (for instance a visual search task—[65]) or should consider using self-reported measures of sleep-related attention bias (e.g. [66]). However, the dot-probe is seen as a standard task to assess attentional bias and Harris and colleagues [23] concluded that the dot-probe (together with the flicker and Posner) appeared one of the most sensitive for group effects.

Apart from this point, there were other limitations to this study. One concerns the timing of the ABM task delivery. In the present study participants were instructed to complete the training between 7 PM and 11 PM. This was designed to target the delivery of the ABM in the lead-up to sleep when biased attention to threat may begin to be problematic, while not requiring participants to complete this task at a time that could interfere with the process of falling asleep. This does however, represent a point of difference from the two earlier studies [29, 30] where the participants were instructed to complete the task immediately before going to bed. As Milkins and colleagues [30] argued, it may be that ABM is most beneficial if it is delivered immediately prior to sleep when the biased attention may be most problematic. Another option is that an ABM training so close to bedtime may lead to increase arousal. This would mean that such a training could better be offered during the day. Second, we based our words on the words used by Clarke and colleagues [29] and these were based on the three-factors of Wicklow and Espie [67]. The study may have been more matched to the target problem if we used only sleep-related words such as those used in Barclay and Ellis [21]. Third, in accordance to the Clark study [29] we used words as stimuli; however, pictorial stimuli may also have been an option for insomnia-related attentional bias and future studies may need to consider other tasks such as a modified Posner. Fourth, we deployed the ABM training online for computers and not on smartphones as Clarke and colleagues [29] did. Other limitations include the self-selected sample and that, due to the online design of the study, we did not have a formal clinical diagnosis of insomnia (although we used DSM-5 guidelines and the ISI ≥ 10 cutoff).

When weighing these strengths and limitations, it is difficult to give a definite answer to the question whether our study should be seen as a failed manipulation of attentional bias or as evidence against the hypotheses. Regarding the failed manipulation, we think the most serious limitation of our study (and indeed many past research) is the unreliable dot-probe task. We believe it is critical to replicate our study with a measure capable of reliably assessing attentions bias. If such study, again, does not observe (changes in) attentional bias, it can be concluded that ABM trainings are not (yet) able to change sleep-related attentional bias. If on the other hand, these changes on attentional bias are observed, but do not lead to symptom changes, this would have strong implications for the theoretical and clinical role of attentional bias (training) in insomnia research. Specifically, such a finding would suggest that attentional bias for sleep-related negative information may be a cognitive correlate of insomnia, but is not causally related to disturbed sleep. As long as no such study with adequate measurements has been reported, we think that no firm conclusions can be drawn on the role of (changing) attentional bias in insomnia complaints. Therefore, the results of the current study clearly provide no evidence for the clinical efficacy of ABM for insomnia and suggest caution in interpreting the potential applied benefits of such an intervention approach on the basis of other recent findings [29, 30]. Given the outcome of the present study, the possibility that these past results could potentially represent a Type-I error will remain until it can be shown that such effects can be replicated.

## Supporting information

S1 Table Word sets in Dutch and translated into English. (PDF)

S2 Table Baseline, Posttest, and Follow-up Scores and Cohen’s d Effect Sizes for the remaining variables for the ABM and Placebo Conditions. (PDF)

S3 Table Completer sample: Baseline, Posttest, and Follow-up Scores and Cohen’s d Effect Sizes for the Completers of the ABM and Placebo Conditions. (PDF)

S4 Table Multilevel regression analyses effects for time, condition, and time × condition. (PDF)

S1 File CONSORT Checklist. (PDF)

S2 File Study protocol. (PDF)
