# Supplementary material for: Attentional bias modification training for insomnia: A double-blind placebo controlled randomized trial
Source: PLoS One. 2017 Apr 19;12(4):e0174531. doi: 10.1371/journal.pone.0174531 (PMC5396867; doi:10.1371/journal.pone.0174531)
Supplement: S1 Table — (PDF) [file pone.0174531.s001.pdf]

S1 Table - *Word sets in Dutch and translated into English.*

Sleep related words	Frequency (in Dutch language)	Neutral words	Frequency (in Dutch language)
**Set A**			
1. Sloom – *lazy/slow*	0.91	Mails - *Mails*	0.91
2. Stress - *Stress*	13.86	Nut - *use*	13.86
3. Klok - *Clock*	23.90	Bril - *Glasses*	24.49
4. Woelen - *Tossing*	0.71	Vloeide - *Flowed*	0.71
5. Boos - *Angry*	105.79	Noem - *Mention*	105.24
6. Moe - *Tired*	89.94	Reis - *Journey*	90.37
7. Bed - *Bed*	239.93	Stel - *Couple*	214.94
8. Wakker - *Awake*	201.37	Nummer - *Number*	202.70
9. Conflict - *Conflict*	4.96	Stopten - *Stopped*	4.96
10. Alarm - *Alarm*	34.78	Koffer - *Suitcase*	33.87
11. Slapen - *Sleeping*	209.22	Leren - *(to) learn*	211.23
12. Wegdromen - *Daydreaming*	0.11	Zwemkleren - *Swimming clothes*	0.11
13. Slaapstoornis - *Sleep disorder*	0.14	Stempatroon - *voicepattern*	0.14
14. Slaperigheid - *Sleepiness*	0.07	Slimmeriken - *Smart guys*	0.64
15. Lamlendig - *Drained/lifeless*	1.23	Achterlicht - *Taillight*	1.23
16. Inslapen - *Falling asleep*	1.85	Postzegels - *Postage stamps*	1.85
17. Radeloos - *Distraught*	2.20	Reservoir - *Reservoir*	2.2
18. Wanhopig - *Desperate*	17.86	Gezichten - *Faces*	17.86
19. Gedachten - *Thoughts*	75.76	Herinneren - *Remember*	76.38

20. Ellendig - *Miserable*	6.01	Degelijk - *Decent*	6.01
21. Slaapkamer - *Bedroom*	30.83	Badkamer - *Bathroom*	30.41
22. Gespannen - *Tense*	17.81	Zekerheid - *Certainty*	17.77
23. Irrationeel - *Irrational*	1.23	Getolereerd - *Tolerated*	1.23
24. Lijden - *Suffering*	35.88	Broeder - *Brother*	35.90

Set B

1. Traag - *Slow*	9.79	Spuit - *Squirt*	9.79
2. Futloos - *Spiritless*	0.14	Lesgeld - *Tuition*	0.32
3. Tijd - *Time*	898.46	Gedaan - *Done*	883.04
4. Lui - *Lazy*	47.68	Vlak - *Flat*	47.73
5. Angst - *Anxiety*	69.34	Kalm - *Calm*	69.43
6. Ziek - *Sick*	129.20	Woord - *Word*	129.09
7. Nacht - *Night*	204.44	Plaats - *Place*	213.38
8. Wekker - *Alarm clock*	3.52	Stuiver - *Penny*	3.50
9. Paniek - *Panic*	39.86	Landen - *Countries*	39.74
10. Alert - *Alert*	6.01	Intact - *Intact*	6.04
11. Liggen - *Lie down*	194.83	Mogen - *To be allowed*	200.83
12. Slapeloos - *Sleepless*	0.11	Schoolfoto - *School picture*	0.11
13. Slaapproblemen - *Sleeping problems*	0.53	Smakelijke - *Tasty*	0.53
14. Slaappillen - *Sleeping pills*	3.06	Enveloppe - *Envelope*	3.06
15. Vermoeidheid - *Tiredness*	1.23	Goudstukken - *Gold pieces*	1.23
16. Verslapen - *To oversleep*	1.92	Rozijnen - *Raisins*	1.92

17. Rusteloos - *Restless*	1.92	Symbolisch - *Symbolic*	1.92
18. Rampzalig - *Disastrous*	2.22	Opheffen - *Cancelling*	2.22
19. Piekeren - *Worrying*	3.25	Minimum - *Minimum*	3.25
20. Hopeloos - *Hopeless*	11.85	Finale - *Finals*	11.85
21. Nachtmerries - *Nightmares*	13.01	Maatschappij - *Community*	12.99
22. Uitgeput - *Exhausted*	13.01	Doorgeven - *Passing*	13.01
23. Geïrriteerd - *Irritated*	2.56	Internet - *Internet*	17.20
24. Ongelukkig - *Unhappy*	18.20	Ondertussen - *Meanwhile*	18.13
**Practice set**			
1. Accent - *Accent*		Vierkant - *Square*	
2. Stoep - *Sidewalk*		Lijn - *Line*	

Note. Sleep related words were always paired with the neutral word that it is paired with in this table
