# Supplementary material for: Attentional bias modification training for insomnia: A double-blind placebo controlled randomized trial
Source: PLoS One. 2017 Apr 19;12(4):e0174531. doi: 10.1371/journal.pone.0174531 (PMC5396867; doi:10.1371/journal.pone.0174531)
Supplement: S2 Table — (PDF) [file pone.0174531.s002.pdf]

S2 Table

Baseline, Posttest, and Follow-up Scores and Cohen's *d* Effect Sizes for the remaining variables for the ABM and Placebo Conditions

Study variable	Condition	Baseline Mean (*SD*)	Posttest Mean (*SD*)	Follow-up Mean (*SD*)	Cohen's *d*			
					Within-group baseline-posttest	Within-group baseline-follow-up	Between-group postttest	Between-group follow-up
Sleep problems (PSQI)	ABM	12.44 (2.89)	11.13 (3.56)	10.82 (3.78)	-0.40	-0.48	0.04	0.10
	Placebo	12.70 (2.69)	11.51 (3.08)	11.44 (3.54)	-0.41	-0.40		
Beliefs (DBAS)	ABM	5.12 (1.56)	4.91 (1.52)	5.05 (1.91)	-0.14	-0.04	0.08	0.10
	Placebo	5.25 (1.32)	5.14 (1.62)	5.33 (1.61)	-0.07	0.05		
Total sleep time (TST)	ABM	339.68 (86.38)	355.22 (61.39)	-	0.21	-	0.03	-
	Placebo	335.92 (78.44)	353.34 (73.76)		0.23			
Wake after sleep onset (WASO)	ABM	46.85 (37.82)	48.58 (23.85)	-	0.05	-	0.06	-
	Placebo	56.84 (42.79)	60.93 (37.51)		0.10			
Terminal wakefulness (TWAK)	ABM	54.91 (30.18)	56.21 (31.82)	-	0.04	-	0.35	-
	Placebo	57.79 (34.23)	48.62 (30.30)		-0.28			
Number of awakenings (NWAK)	ABM	1.33 (1.33)	2.11 (1.96)	-	0.53	-	0.28	-
	Placebo	1.48 (1.21)	1.85 (1.46)		0.28			
Sleep quality (SQ)	ABM	2.85 (0.54)	3.15 (0.63)	-	0.51	-	0.18	-
	Placebo	2.81 (0.48)	2.99 (0.55)		0.17			
Sleep worry – diary (APSQ)	ABM	26.65 (8.38)	25.61 (10.10)	-	-0.11	-	0.20	-
	Placebo	28.22 (8.58)	28.78 (8.51)		0.07			

Note. Missing data are imputed with 10 separate datasets on the basis of predictive mean matching.
