# Supplementary material for: Attentional bias modification training for insomnia: A double-blind placebo controlled randomized trial
Source: PLoS One. 2017 Apr 19;12(4):e0174531. doi: 10.1371/journal.pone.0174531 (PMC5396867; doi:10.1371/journal.pone.0174531)
Supplement: S3 Table — (PDF) [file pone.0174531.s003.pdf]

S3 Table

Completer sample: Baseline, Posttest, and Follow-up Scores and Cohen's *d* Effect Sizes for the Completers of the ABM and Placebo Conditions

Study variable	Condition	Baseline Mean (*SD*)	Posttest Mean (*SD*)	Follow-up Mean (*SD*)	Cohen's *d*			
					Within-group baseline-posttest	Within-group baseline-follow-up	Between-group postttest	Between-group follow-up
Insomnia Severity (ISI)	ABM	14.60 (3.01)	13.17 (3.73)	12.86 (3.54)	-0.42	-0.53	0.05	0.23
	Placebo	14.61 (3.11)	13.37 (3.39)	13.59 (3.78)	-0.38	-0.30		
Depressive symptoms (CESD)	ABM	14.74 (6.68)	16.12 (7.70)	14.43 (6.88)	0.21	-0.05	0.10	0.28
	Placebo	15.56 (5.31)	17.58 (7.92)	17.10 (8.34)	0.30	0.22		
Anxiety (HADS)	ABM	6.07 (3.09)	5.40 (3.67)	5.10 (2.97)	-0.20	-0.32	0.29	0.26
	Placebo	5.76 (3.21)	5.89 (3.17)	5.56 (3.52)	0.02	-0.03		
Sleep worry (APSQ)	ABM	35.90 (7.00)	30.66 (9.30)	30.80 (8.03)	-0.64	-0.68	0.27	0.16
	Placebo	35.85 (6.67)	32.76 (8.76)	32.15 (9.44)	-0.40	-0.45		
Sleep onset latency (SOL)	ABM	41.56 (36.11)	41.87 (34.22)	-	0.01	-	0.03	-
	Placebo	37.90 (29.39)	39.10 (32.35)		0.05			
Sleep efficiency (SE)	ABM	69.15 (14.93)	69.65 (11.54)	-	0.04	-	0.04	-
	Placebo	69.12 (14.82)	70.08 (15.33)		0.06			
Attention bias	ABM	4.53 (30.66)	4.68 (22.69)	-	0.01	-	0.04	-
	Placebo	1.43 (23.57)	0.23 (22.58)		-0.05			

Note. Completers are participants that completed six (out of eight) or more trainings. Missing data are imputed with 10 separate datasets on the basis of predictive mean matching.

S3 Table - Continued

Study variable	Condition	Baseline Mean (*SD*)	Posttest Mean (*SD*)	Follow-up Mean (*SD*)	Cohen's *d*			
					Within-group baseline-posttest	Within-group baseline-follow-up	Between-group postttest	Between-group follow-up
Sleep problems (PSQI)	ABM	12.59 (2.90)	11.28 (3.39)	11.33 (2.80)	-0.42	-0.44	0.02	0.03
	Placebo	12.67 (2.78)	11.43 (2.96)	11.50 (3.29)	-0.43	-0.38		
Beliefs (DBAS)	ABM	5.16 (1.49)	4.96 (1.52)	5.00 (1.65)	-0.14	-0.10	0.15	0.13
	Placebo	5.25 (1.24)	5.22 (1.51)	5.25 (1.53)	-0.02	0.00		
Total sleep time (TST)	ABM	342.89 (80.77)	354.22 (65.59)	-	0.15	-	0.16	-
	Placebo	333.26 (75.35)	354.09 (78.57)		0.27			
Wake after sleep onset (WASO)	ABM	47.55 (38.40)	49.43 (25.04)	-	0.06	-	0.10	-
	Placebo	54.89 (41.41)	60.50 (39.93)		0.14			
Terminal wakefulness (TWAK)	ABM	57.37 (31.29)	56.45 (33.96)	-	-0.03	-	0.32	-
	Placebo	58.77 (35.14)	48.08 (32.23)		-0.32			
Number of awakenings (NWAK)	ABM	1.38 (1.15)	1.89 (1.12)	-	0.49	-	0.03	-
	Placebo	1.27 (0.95)	1.81 (1.46)		0.44			
Sleep quality (SQ)	ABM	2.79 (0.50)	3.06 (0.48)	-	0.54	-	0.21	-
	Placebo	2.84 (0.48)	2.98 (0.53)		0.13			
Sleep worry – diary (APSQ)	ABM	27.02 (8.42)	26.17 (8.17)	-	-0.13	-	0.18	-
	Placebo	28.54 (8.41)	28.80 (9.10)		0.03			
