# Supplementary material for: Attentional bias modification training for insomnia: A double-blind placebo controlled randomized trial
Source: PLoS One. 2017 Apr 19;12(4):e0174531. doi: 10.1371/journal.pone.0174531 (PMC5396867; doi:10.1371/journal.pone.0174531)
Supplement: S4 Table — (PDF) [file pone.0174531.s004.pdf]

S4 Table – Multilevel regression analyses effects for time, condition, and time × condition

	ISI		PSQI		CESD		HADS-A		DBAS		APSQ	
	b	*(SE)*	*b*	*(SE)*	*b*	*(SE)*	*b*	*(SE)*	*b*	*(SE)*	*b*	*(SE)*
ABM training												
Constant	14.58	(0.38)***	12.54	(0.35)***	14.53	(0.72)***	6.34	(0.33)***	5.16	(0.18)***	35.61	(0.96)***
Post	-1.69	(0.38)***	-1.28	(0.36)***	1.15	(0.75)ns	-0.74	(0.36)*	-0.16	(0.13)ns	-5.00	(0.93)***
Follow-up	-1.86	(0.39)***	-1.39	(0.37)***	-0.59	(0.78)ns	-1.08	(0.34)**	-0.18	(0.13)ns	-5.09	(0.95)***
ABM	0.16	(0.53)ns	0.08	(0.49)ns	1.18	(1.01)ns	-0.67	(0.46)ns	0.08	(0.25)ns	0.50	(1.35)ns
ABM x post	0.22	(0.52)ns	-0.01	(0.50)ns	0.54	(1.03)ns	0.79	(0.46)ns	0.07	(0.18)ns	1.13	(1.28)ns
ABM x follow-up	0.54	(0.53)ns	0.19	(0.50)ns	1.52	(1.08)ns	0.83	(0.47)ns	0.17	(0.18)ns	1.14	(1.30)ns
Placebo training												
Constant	14.74	(0.37)***	12.62	(0.34)***	15.70	(0.70)***	5.67	(0.32)***	5.24	(0.17)***	36.11	(0.94)***
Post	-1.48	(0.36)***	-1.29	(0.34)***	1.69	(0.71)*	0.05	(0.32)ns	-0.09	(0.12)ns	-3.88	(0.89)***
Follow-up	-1.31	(0.36)***	-1.21	(0.34)***	0.93	(0.73)ns	-0.25	(0.32)ns	-0.00	(0.13)ns	-3.95	(0.89)***

Note. The non-standardized regression coefficients are indicative of the relative change between pre- and post-test; * = P < 0.05; ** = P < 0.01; *** = P < 0.001; if the variables that were associated with non-response (PSQI; terminal wakefulness; amount of training sessions completed. CESD; HADS-A; number of awakening) correlated with the dependent variable, these variables were added as covariate. ISI = Insomnia Severity Index; PSQI = Pittsburgh Sleep Quality Index; CESD = Centre of Epidemiological Studies Depression scale; HADS-A = Hospital Anxiety and Depression Scale; DBAS = Dysfunctional Belief and Attitudes about Sleep scale; APSQ = Anxiety and Preoccupation about Sleep Questionnaire.

S4 Table – Continued

	TST		SE		SOL		WASO		TWAK	
	b	*(SE)*	*b*	*(SE)*	*b*	*(SE)*	*b*	*(SE)*	*b*	*(SE)*
<u>ABM training</u>										
Constant	337.09	(7.81)***	68.39	(1.34)***	41.84	(3.17)***	47.24	(4.41)***	55.31	(4.02)***
Post	18.11	(7.98)*	1.78	(1.47)^{ns}	0.28	(3.42)^{ns}	0.06	(5.24)^{ns}	-1.97	(4.04)^{ns}
ABM	-4.49	(11.02)^{ns}	-0.16	(1.89)^{ns}	-3.97	(4.47)^{ns}	8.90	(6.23)^{ns}	2.10	(5.67)^{ns}
ABM x post	3.47	(11.14)^{ns}	-0.27	(2.06)^{ns}	-0.49	(4.77)^{ns}	4.84	(7.34)^{ns}	-7.38	(5.60)^{ns}
<u>Placebo training</u>										
Constant	332.60	(7.80)***	68.24	(1.34)***	37.86	(3.15)***	56.13	(4.40)***	57.41	(3.99)***
Post	21.58	(7.79)**	1.51	(1.44)^{ns}	-0.22	(3.33)^{ns}	4.91	(5.13)^{ns}	-9.34	(3.88)*

Note. The non-standardized regression coefficients are indicative of the relative change between pre- and post-test; * = P < 0.05; ** = P < 0.01; *** = P < 0.001; if the variables that were associated with non-response (PSQI; terminal wakefulness; amount of training sessions completed. CESD; HADS-A; number of awakening) correlated with the dependent variable, these variables were added as covariate. TST = Total sleep time; SE = Sleep efficiency; SOL = Sleep onset latency; WASO = Wake after sleep onset; TWAK = Terminal wakefulness.

S4 Table – Continued

	NWAK		SQ		APSQ diary		Attention bias	
	b	*(SE)*	*b*	*(SE)*	*b*	*(SE)*	*b*	*(SE)*
<u>ABM training</u>								
Constant	1.42	(0.14)***	2.84	(0.06)***	26.69	(1.00)***	4.68	(3.42)ns
Post	0.44	(0.12)***	0.264	(0.26)***	-1.01	(0.79)ns	-0.05	(5.37)ns
ABM	0.04	(0.20)ns	-0.02	(0.09)ns	1.54	(1.42)ns	-6.08	(4.78)ns
ABM x post	0.07	(0.17)ns	-0.11	(0.10)ns	1.32	(1.10)ns	-0.34	(7.47)ns
<u>Placebo training</u>								
Constant	1.46	(0.14)***	2.83	(0.06)***	28.23	(1.00)***	-1.40	(3.33)ns
Post	0.51	(0.12)***	0.15	(0.07)*	0.31	(0.77)ns	0.29	(5.20)ns

Note. The non-standardized regression coefficients are indicative of the relative change between pre- and post-test; * = P < 0.05; ** = P < 0.01; *** = P < 0.001; if the variables that were associated with non-response (PSQI; terminal wakefulness; amount of training sessions completed. CESD; HADS-A; number of awakening) correlated with the dependent variable, these variables were added as covariate. NWAK = Number of nightly awakenings; SQ = Sleep Quality; APSQ = Anxiety and Preoccupation about Sleep Questionnaire – diary version.
