# Supplementary material for: Attentional bias modification training for insomnia: A double-blind placebo controlled randomized trial
Source: PLoS One. 2017 Apr 19;12(4):e0174531. doi: 10.1371/journal.pone.0174531 (PMC5396867; doi:10.1371/journal.pone.0174531)
Supplement: S2 File — (PDF) [file pone.0174531.s006.pdf]

S2 File. Study protocol.

Approval…………………………………………………………………………… Page 2

Study protocol……………………………………………………………………. Page 3-7

Translated parts…………………………………………………………………. Page 8-9

UNIVERSITEIT VAN AMSTERDAM

LAB Ethics Review Board
Faculty of Social and Behavioral Sciences

Department of Psychology

Nieuwe Achtergracht 129B
1018 WS Amsterdam

The Netherlands
T +31 (0)20 525 6686
http://www.uva.nl

http://ethiek.fmg.uva.nl

Date
05-08-2016

E-mail
w.p.m.vandenwildenberg@uva.nl

Subject
Approval notice

**Project title**	Attention bias modificatie training: een nieuwe behandeling voor insomnie?			
**Project#**	2015-CP-3862			
**Project collaborators**	owner	Jaap Lancee	0205258609	J.Lancee@uva.nl
	erb consultant	Arnold van Emmerik		A.A.P.vanEmmerik@uva.nl

The research project titled *"Attention bias modificatie training: een nieuwe behandeling voor insomnie?"* (filed as 2015-CP-3862) submitted by Jaap Lancee with the guidelines formulated by the Ethics Review Board of the Faculty of Social and Behavioral Sciences, University of Amsterdam, The Netherlands, and has been approved by the aforementioned Ethics Review Board on March 17th, 2015.

Sincerely,

Wery P.M. van den Wildenberg, PhD

Chair Ethics Review Board (FMG-UvA)
University of Amsterdam

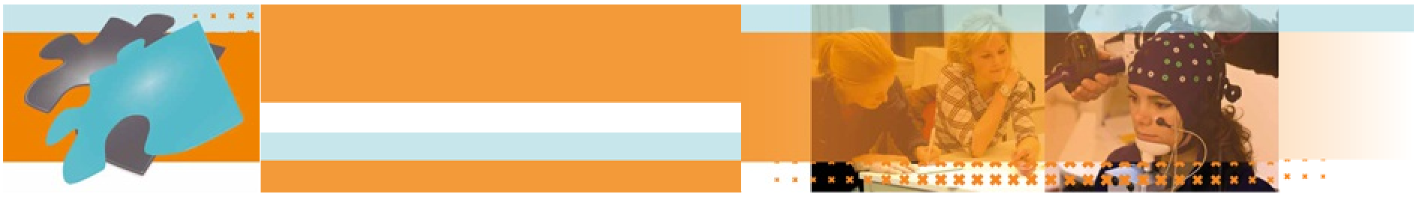

Attention bias modificatie training: een nieuwe behandeling voor insomnie?

Project details

**ERB number**	2015-CP-3862
**ID**	3862
**Title**	Attention bias modificatie training: een nieuwe behandeling voor insomnie?
**Department**	Clinical Psychology
**Status**	ERB approved
**Created**	2015-03-17 10:43:28
**Modified**	2016-08-04 00:00:37

Project collaborators

Jaap Lancee	Owner	J.Lancee@uva.nl	0205258609

General

Responsible researcher

Jaap Lancee

Who conducts the research?

Samya Yasiney en Bas Brendel

Research location

Universiteit van Amsterdam

Brief project description

Cognitieve gedragstherapie is de meest effectieve behandeling voor insomnie. Deze therapie richt zich echter nog niet zo zeer op selectieve aandacht (attention bias) dat ook een rol lijkt te spelen bij mensen met insomnie. In dit project gaan wij onderzoeken of attention bias modifciatie training ook werkt voor het verminderen van insomnie symptomen. Wij verwachten dat een dergelijke training voor insomnie werkt, omdat 1) bij angst ook is gebleken dat angstsymptomen verminderen door een attention bias modificatie training en 2) een pilot in Australië die hetzelfde onderzocht positieve resultaten heeft opgeleverd. Er worden 140 participanten geworven die worden gerandomiseerd in: 1) attention bias modificatie training; 2) controle training. De training duurt in totaal 2 weken. Om de effectiviteit van de training te bepalen vullen de participanten vragenlijsten in om slaapsymptomen te meten en voeren ze een dot-probe taak uit om attention bias te meten direct voor, direct na en een week na de training. Ook wordt er een week voor, tijdens en een week na de training iedere dag een slaapdagboek ingevuld door de participanten. De primaire uitkomstmaat is de Insomnia Severity Index.

Expected duration of the project

3 maanden

Expected number of participants

140

This project is comparable with the following submitted project(number)

3152

Scientific integrity checklist

Who will store and inspect the data?

Jaap Lancee, Samya Yasiney, Bas Brendel

When will data collection start (approximately)?

2015-01-05

When will the data collection be completed?

2017-02-13

Is the study exploratory or confirmatory?

confirmatory (also in case of pre-registration)

Ethics Checklist

A1. When classifying the research as Medical vs. Non-medical, does it comply with A1, meaning it can be listed under category D (see also Appendix 1, 2.4)?

Yes, it falls into category D

A2. Are consenting adults selected, as described in A2?

Yes, Category 3: Participants are recruited based on specific individual features

A3. Are participants free to decide to participate and to stop for whatever reason, as listed under A3?

Yes

A4. Are participants subjected to a screening procedure to reduce the risks for adverse effects, as listed under A4?

No,I explain in a comment why

Mensen moeten voldoen aan onze criteria voor insomnie. Daarnaast mogen deelnemers met psychose/schizofrenie, slaapapneu of een huidige depressieve episode niet deelnemen.

A5. Is there a risk for chance incidents that should be reported to the participant, as listed under A5?

No, the method precludes chance incidents

A6. Are participants fully informed before participating, and do they sign a consent form, as listed under A6?

Yes, please submit the information letter and the consent form as attachment

A7. Is participant privacy and anonymity guaranteed, as listed under A7?

Yes

A8. In case of deception, does the procedure comply with the conditions listed under A8? (full disclosure concerning risks, accurate debriefing)?

There is no deception

A9. Is there a risk that a substantial number of participants will drop out because the research is considered to be discomforting, as listed under A9?

No

B0. Does the research fully comply with the guidelines for Standard Research?

No, I specify in a comment why not

Deelnemers krijgen een training die er op gericht is om de klachten van slapeloosheid te verminderen. Daarbij zijn er 4 trainingen per week en is er een dagelijks slaapdagboek dat ingevuld moet worden.

Attachments

Add a concise research description (max 1 A4) and any other relevant documents.

1. Onderzoeksbeschrijving.docx

Scientific integrity follow up

Did you complete the study?

I completed the study, but I am still trying to publish it (option to enter a new end date)

Project history

2016-08-05 08:21:47 Set new project end date to: 2017-02-13 [by Jaap Lancee]

2016-08-04 00:00:37 Scientific integrity follow up [by Unknown]

According to the information you supplied this project is completed or will be soon. Please answer some evaluation questions or update the study end date in the Scientific integrity follow up section of the project

---

2016-08-03 15:20:37 Project approved [by Wery van den Wildenberg]

beste Jaap,

Ik heb op een "approval" button geclickt; wellicht is de SI-melding nu verdwenen.

met groeten,

wery

From: Lancee, Jaap [mailto:J.Lancee@uva.nl]
Sent: 3 August, 2016 10:57
To: Wildenberg, Wery van den <W.P.M.vandenWildenberg@uva.nl>
Subject: Vraag over 2015-CP-3862

Beste Wery,

Arnold heeft mij naar jou doorverwezen voor een vraag over 2015-CP-3862.

Ik wil het artikel wat gaat over dit onderzoek indienen bij PLOS ONE. Echter bij de status staat nu 'submitted for SI review'. Dat staat niet zo netjes als ik er een pdf van maak. Dit komt doordat ik toentertijd bij de SI review heb ingevuld 'I started the study but did not complete it (the project will close unfinished)'. Dit klopte toen ook maar is nu niet meer waar. Ik zou het nu graag aanpassen zodat ook de status van het project weer verandert naar ERB approved. Weet jij hoe ik dat kan doen?

Alvast bedankt!

Groeten,

Jaap

Jaap Lancee, PhD

Department of Clinical Psychology, University of Amsterdam

Nieuwe Achtergracht 129B | Kamer G1.41

http://www.uva.nl/over-de-uva/organisatie/medewerkers/content/l/a/j.lancee/j.lancee.html

www.insomnie.nl; www.nachtmerries.org; www.allesoverdromen.nl

----

2015-03-29 14:01:10 Jaap Lancee [by Jaap Lancee]

Project submitted for SI evaluation

2015-03-29 00:01:59 Automated system [by Unknown]

Request Scientific integrity follow up notification send

2015-03-17 10:52:50 Arnold van Emmerik [by Arnold van Emmerik]

Project approved

2015-03-17 10:52:50 Arnold van Emmerik [by Arnold van Emmerik]

Beste Jaap,

Bij deze heb ik de aanvraag weer gesloten, aangenomen dat je de gewenste informatie inmiddels hebt toegevoegd.

Regards,

Arnold van Emmerik,
Member of the Ethics Review Board.

2015-03-17 10:43:28 Jaap Lancee [by Jaap Lancee]

Date data ready updated to: 29-03-2015

2015-03-17 10:43:28 Jaap Lancee [by Jaap Lancee]

Changed project submitted for ERB evaluation

2015-03-17 09:21:36 Arnold van Emmerik [by Arnold van Emmerik]

Request for additional information

2015-03-17 09:21:36 Arnold van Emmerik [by Arnold van Emmerik]

Beste Jaap,
Op jouw verzoek, open ik hierbij deze aanvraag zodat je er informatie aan kunt toevoegen.
MVG, Arnold

Dear Colleague,

I hereby acknowledge receipt of your project, archived as 2014-CP-3862.
Please use this file number in future correspondence.

In order to review your project, you are kindly requested to provide the following additional information or clarification:

1.
2.
3.

To submit the above-mentioned information, your project appears in the list of "Open Projects" under "My Projects".
Here you can modify the general project information as well as the required file attachments and resubmit your project for review.

Please keep in mind that formal approval by the Ethics Review Board is mandatory before starting your research.

Regards,

Arnold van Emmerik,

Member of the Ethics Review Board

2014-11-01 16:08:58 Arnold van Emmerik [by Arnold van Emmerik]

Project approved

2014-11-01 16:08:58 Arnold van Emmerik [by Arnold van Emmerik]

Dear Colleague,

I hereby acknowledge receipt of your project, archived as 2014-CP-3862.
Please use this file number in future correspondence.

Your project has been reviewed and is hereby approved.

Modifications of the concerning project should be submitted to the Ethics Review Board for evaluation.

Regards,

Arnold van Emmerik,
Member of the Ethics Review Board.

2014-10-24 14:03:52 Jaap Lancee [by Jaap Lancee]

Date data ready set to: 29-03-2015

2014-10-24 14:03:52 Jaap Lancee [by Jaap Lancee]

Project submitted for ERB evaluation

2014-10-21 17:01:06 delegate (s.yasiney@hotmail.com) [by Jaap Lancee]

Delegate project submitted

2014-10-19 12:46:35 delegate (s.yasiney@hotmail.com) [by Jaap Lancee]

Project created

(google)Translated parts of ERB approval

Brief description of the project

Cognitive behavioral therapy is the most effective treatment for insomnia. This therapy, however, focuses yet not so much selective attention (attention bias), which also appears to play a role in people with insomnia. In this project we will investigate whether attention bias modifciatie training also works to reduce insomnia symptoms. We expect that such a training works for insomnia, because 1) in anxiety also showed that reducing anxiety symptoms by an attention bias modification training, and 2) a pilot in Australia which has yielded the same results tested positive. 140 participants are being recruited to be randomized to: 1) attention bias modification training; 2) control training. The training lasts for two weeks. To determine the effectiveness of the training the participants completed questionnaires to measure sleep symptoms and perform a dot-probe task to attention bias to measure immediately before, immediately after and one week after training. A week is for a sleep diary filled out by the participants during each day and one week after training. The primary outcome measure is the Insomnia Severity Index.

Concise description of the project

Subjects are recruited via www.insomnie.nl. If participants are interested can fill out an online questionnaire. they advance to give this online questionnaire digitally informed consent. Once they have done this they completed questionnaires about demographic and general data, chronic insomnia (Insomnia Severity Index (ISI), Pittsburgh Sleeping Quality Index (PSQI), Anxiety and preoccupation about Sleep Questionnaire (APSQ) and depression (Beck Depression Inventory; BDI).

Inclusion criteria were: insomnia according to DSM-5, Insomnia Severity Index> 10, 18+, disposal valid e-mail address, Internet and a computer.

Exclusion criteria: sleep apnea, starting psychotherapy in the past six months, medication, alcohol or drug abuse, symptoms suggestive of a current depressive episode (BDI - cutoff 17; Carney et al., 2009), diagnosis of psychosis / schizophrenia, working in shifts.

Participants will be screened online at the above exclusion criteria. If participants have symptoms suggestive of a depressive episode or a diagnosis of psychosis / schizophrenia then incorporate them advised to contact their GP.

The subjects are randomly assigned to 1) attention bias modification (ABM) training or 2) control training. The randomization is carried out by the Lotus program. Lotus can conceal the distribution of the test subjects for the investigators and participants, and thereby the double-blind study can be carried out. Both conditions last 4 weeks. In the first week the above screening is done and the participants keep a sleep diary. This sleep diary the participants throughout the four weeks. In the

second week the training begins. For the training, the level of attention bias is measured. Thereafter, the ABM follows training. In the active training the probe follows after 100% neutral words, so that attention is drawn away from the sleep-related stimuli. In the control group, the probe 50% after neutral words and 50% after sleep-related words and nothing is being trained. The participants will be trained two weeks, four times a week. Each course is offered nightly online and takes about 15 minutes. After the last training session (or the day after the last training) is again decreased a normal dot-probe task to measure the attention bias and the ISI, and PSQI APSQ be taken in order to measure the degree of insomnia. In the fourth week is yet to train completed the sleep diary every day without and at the end of the fourth week the follow-up place. The participants then again have a normal dot-probe task measuring attention bias and fill the ISI and PSQI in APSQ. After the training is still offered a free self-help training to sleep all subjects (Lancee, van den Bout, Streets & Spoormaker, 2012).

References:

Carney, C.E., Ulmer, C., Edinger, J.D., Krystal, A.D., & Knauss, F. (2009). Assessing depression symptoms in those with insomnia: An examination of the beck depression inventory second edition (BDI-II). Journal of Psychiatric Research. 43 (5), 576-582.

Lancee, J., van den Bout, J., van Straten, A., & Spoormaker, V. I. (2012). Internet-delivered or mailed self-help treatment for insomnia? A randomized waiting-list controlled trial. *Behaviour research and therapy*, *50*(1), 22-29
